# Synergistic Activity of New Diclofenac and Essential Oils Combinations against Different *Candida* spp.

**DOI:** 10.3390/antibiotics10060688

**Published:** 2021-06-08

**Authors:** Antonio Rosato, Elisabetta Altini, Sabina Sblano, Lara Salvagno, Filippo Maggi, Giuliana de Michele, Alessia Carocci, Maria Lisa Clodoveo, Filomena Corbo, Giuseppe Fracchiolla

**Affiliations:** 1 Department of Pharmacy-Drug Sciences, University of Bari “Aldo Moro”, 70125 Bari, Italy; antonio.rosato@uniba.it (A.R.); e.altini4@studenti.uniba.it (E.A.); sabina.sblano@uniba.it (S.S.); l.salvagno@studenti.uniba.it (L.S.); g.demichele4@studenti.uniba.it (G.d.M.); filomena.corbo@uniba.it (F.C.); giuseppe.fracchiolla@uniba.it (G.F.); 2 School of Pharmacy, University of Camerino, 62032 Camerino, Italy; filippo.maggi@unicam.it; 3 Interdisciplinary Department of Medicine, School of Medicine, University of Bari “Aldo Moro, 70124 Bari, Italy; marialisa.clodoveo@uniba.it

**Keywords:** synergism, *Mentha × piperita*, *Pelargonium graveolens*, *Melaleuca alternifolia*, Diclofenac Sodium Salt

## Abstract

According to recent studies, Non-Steroidal Anti-Inflammatory Drugs (NSAIDs) have shown a good antimicrobial and antifungal activity. Their association with essential oils (EOs) could be useful for the treatment of infections caused by *Candida spp*. The aim of this studyis to evaluate the synergistic antifungal activity of new combinations between Diclofenac Sodium Salt (DSS), a widely used NSAID, with EOs of *Mentha × piperita*, *Pelargonium graveolens* and *Melaleuca alternifolia*. The in-vitro antifungal activity was determined on different *Candida* strains. The determination of the chemical composition of EOs was carried out by gaschromatography-massspectrometry (GC-MS). Susceptibility testing of planktonic cells was performed by using the broth microdilution assay and checkerboard methods. Minimum Inhibitory Concentrations (MIC) of DSS was in a range from 1.02 to 2.05 μg/mL reaching a MIC value of 0.05 μg/mL when combined with *Pelargonium graveolens* (FICI = 0.23–0.35) or *Menthapiperita* (FICI = 0.22–0.30) EOs. These preliminary results show thatthe combination of the EOs with DSS improves the antifungal activity on all the tested *Candida* strains.

## 1. Introduction

Fungal infections should not be underestimated, since their incidence in recent years has increased significantly, especially in immunocompromised patients [1]. Moreover, among all nosocomial fungal infections, those caused by *Candida* spp. are the most difficult to eradicate. Indeed, infections caused by *Candida* spp. can spread and colonize different tissue districts, causing considerable damage up to the compromise of organ functions. Candidiasis and candidemia show a wide spectrum of clinical symptoms of different entities depending on whether they are: superficial infections, affecting the skin and mucous membranes, or of deep and widespread severity [2,3].

Current pharmacological therapies are focused on the use of conventional antifungals such as Amphotericin B [4,5] and synthetic drugs belonging to the azoles class (e.g. Clotrimazole, Ketoconazole, Miconazole) that could also be prescribed in combination with each other depending on the severity of the infection [6]. Recently, the activity of different drugs belonging to other therapeutic classes are being evaluated in the drugs-repositioning strategy as antimicrobials [7]. Drugs such as Promazine (phenothiazine antipsychotic), Promethazine (antihistamine), Methyldopa (centrally acting antidepressant), Dobutamine (sympathomimetic) and Diclofenac (NSAIDs) have shown an interesting antimicrobial activity, and for this reason they have been defined as non-antibiotic drugs [8,9,10,11,12]. According to these results, Diclofenac, also known as (2-[2-(2,6-dichloroanilino)phenyl] acetic acid), one of the more effective cyclooxygenase enzymes (COX) inhibitors, was selected for this research. Indeed, COX inhibitionleads to blockage of prostaglandins (PGs) biosynthesis, contributing to a variety of physiological and pathological functions. Furthermore, current studies show that PGs may play a pivotal role in the regulation of eicosanoids pathway in *Candida* spp. and because of an impairment of their metabolism, the inhibition of PGs synthesis by Diclofenac should cause the fungus death [13,14,15]. Based on this evidence, DSS could be able to reduce the infection, acting as a COX inhibitory agent for the treatment of *Candida* infections.

Recently, research on EOs, whose antifungal activity in traditional medicine has been well documented, has aroused the interest of many researchers. Several recent studies confirmed the potential of these natural products as antifungal agents [16]. Therefore, it is not surprising that EOs are regarded as one of the most promising groups of natural products useful for the development of new broad-spectrum, cheaper, and safer drugs for the treatment of mycosis [17]. Although the precise mechanism of the antifungal action of EOs is not yet explained, the plasma membrane and the cell wall appear to be particularly affected [18,19]. Among EOs, it is already known that *Mentha* x *piperita* L. [20], *Pelargonium graveolens* L’Hér. [21], and *Melaleuca alternifolia* (Maiden & Betche) Cheel [22,23] have antifungal properties. 

Starting from these results, the aim of these preliminary studies is to assess the synergistic effects of a new combination of DSS and EOs against planktonic cells of *Candida* spp., revealing new strategies for the repositioning of this anti-inflammatory drug.

## 2. Results

### 2.1. EOs Chemical Composition

EOs used in this study were analyzed using GC-MS. Their chemical composition is described in Table 1.

About 45 compounds were identified in *P. graveolens* EO corresponding to 89.4% of the whole mixture. This EO was characterized bycitronellol (26.5%), geraniol (11.7%), γ−eudesmol (7.02%), citronellyl formate (6.85%), linalol(4.68%) and *iso*-menthone (4.61%). Other compounds accounted for less than 2%. They were identified as β-bourbonene (1.8%), rose oxide (1.67%), (*E*)-caryophyllene (1.63%), geranyl formate and geranyl tiglate (1.57% both) and 2-phenylethyl tiglate (1.48%).

Pure *M. piperita* EO was characterized for 96% of its composition. Menthol (35.6%) and *neo*-menthol (9.33%) were the major components. Other compounds present in relevant amount were menthone (23.99%), 1,8-cineole (9.70%), *trans*-carene (7.72%) and (*E*)-caryophyllene (2.13%). Several compounds, such as α-pinene, β-pinene, piperitone and pulegone were present in an amount less than 2%, while others are in traces. 

*M. alternifolia* EO was characterized for 82.78%. The major relevant compound was terpinen-4-ol (33.4%). γ−Terpinene accounted for 17.18% of the mixture, followed by aromadendrene (4.41%), ledene (3.93%), and α−terpinolene (3.80%). Several compounds such as α−pinene, *p*-cymene and δ−cadinene comprised about 2% of the mixture, while (*E*)-caryophyllene and isoledene were about 1%. 

### 2.2. Antifungal Activity

In this research, DSS was combined with different EOs to inhibit the fungal growth. The antifungal activity as MIC (minimal inhibitory concentration) of these combinations were reported in Table 2, Table 3 and Table 4. The FIC Index (FICI), a parameter that studies the synergism of two compounds, was also reported. Considering the combination between DSS and EOs, the lowest FICI values are 0.22 for *M. piperita* EO, 0.23 for *P. graveolens* and *M. alternifolia*. It is interesting to note that the concentration in μg/mL of DSS decreases from 2.05 to 0.06 when combined with *M. piperita* EO, to 0.05 in combination with *P. graveolens* EO and to0.10 in association with *M. alternifolia* EO (Table 2, Table 3 and Table 4).

## 3. Discussion 

The emergence and development of antifungal drug resistance in *Candida* spp. constitute a serious concern. A successful combination of therapy for the treatment of fungal infectious diseases can achieve broader antifungal coverage and potentially reduce acquired resistance. The combination of repositioned drugs with EOs is also an interesting approach for the rapid identification of new therapies to treat acute infections. Several studies demonstrated that NSAIDs exhibited antifungal activity against *Candida* species alone or in combination with antifungal agents [29,30]. The antifungal activity of NSAIDs is conceivably related to the inhibition of the COX leading to decrease the levels of prostaglandins that are known to be produced by *Candida* spp. Among NSAIDs, DSS is an anti-inflammatory drug whose activity on eukaryotic fungal cells was likely determined by an impairment of PGs metabolism. In fact, DSS causes an inhibition of prostaglandin synthesis. Due to their potential therapeutic effects, EOs are widely used as alternative antimicrobial agents against various infections.

Our previous studies on EOs showed their synergy with some commercially available antibiotics and demonstrated the effectiveness of these associations by proposing the possibility of a new therapeutic use [31,32,33,34,35]. 

In the present study, we reported the effect of DSS in combination with EOs of *M. piperita*, *P. graveolens* and *M. alternifolia* on the growth of *Candida* spp. from ATCC collection and clinical isolation. As highlighted in our in-vitro assays, *Candida* spp. planktonic cells have shown their sensitivity to the compounds tested, both individually and in combination. Table 2, Table 3 and Table 4 show the antifungal activity against *Candida* spp. of DSS alone or in combination with EOs tested. The results obtained allow us to confirm the synergistic effect between DSS and the EOs under study. Indeed, the data clearly show a significant reduction in the active concentration of NSAID when used in association with EOs for all fungal strains tested. It is noteworthy that, when tested in association with *M. piperita* EO, the MIC value for DSS is reduced from 1.02 μg/mL to 0.05 μg/mL and from 1.02 μg/mL to 0.06 μg/mL for *C. parapsilosis* 11A13 and *C. krusei* ATCC 6258, respectively. With regard to the association with *P. graveolens* EO, it is particularly noteworthy that the MIC value of DSS is reduced from 1.02 μg/mL to 0.05 μg/mL for *C. parapsilosis* 910. Interestingly, the MIC value of DSS is reduced from 1.02 μg/mL to 0.05 μg/mL for *C. parapsilosis* 11A13 and *C. parapsilosis* 910, when tested in association with *M. alternifolia* EO. These promising results obtained allow us to confirm the synergistic effect between DSS and the EOs under study. This activity should be ascribed to the presence of fundamental active compounds in EOs such as terpene alcohols and hydrocarbons acting in association with DSS. The mechanism of action is conceivably multifactorial, deriving from the complex synergy of the components. As reported in several scientific works, the synergy of EO could be explained by their ability to disrupt the permeability barrier of the microbial plasma membrane [18,19]. This disruption could conceivably facilitate the entry of DSS into the microbial cell, thus interacting with the COX systems and ultimately causing its antifungal action.

## 4. Material and Methods 

### 4.1. Material 

The pure *M. piperita* EO (LOT F011023, 10/2023), the pure *P. graveolens* EO (LOT F810074, 07/2022) and the pure *M. alternifolia* EO (F911010, 04/2024) were provided by Puressentiel Italia (Milano, Italy) and were stored in a brown glass bottle at the temperature of 0–4 °C until the testing analysis or microbiological assays. The DSS was purchased from Farmalabor (Canosa di Puglia—Bari, Italy). Solvents (analytical grade), *n*-alkanes standard mixture C7–C40and all standard compounds (17, 24–26, 30, 34 and 68 listed in Table 1) used to compare GC-MS analyses were purchased from Supelco Sigma-Aldrich S.r.l. (Milano, Italy). Filters were supplied by Agilent Technologies Italia S.p.a (Milano, Italy).The culture media used are Sabouraud 2% dextrose broth (Oxoid, Italy) and Yeast Malt Broth (Oxoid, Italy).The antifungal activity was tested against many fungal strains and include different strains belonging to the American Type Culture Collection (ATCC, Rockville, MD, USA) or derived from clinical isolation. Strains from the ATCC were *C. albicans* (ATCC 10231), *C. albicans* (ATCC 90028), *C. glabrata* (ATCC 15126)*, C. tropicalis* (ATCC 750)*, C. kefyr* (ATCC 204093)*,* *C. krusei* (ATCC 6258). All the isolates were from patients admitted to the intensive care unit of the Department of Biomedical Science and Human Oncology, University of Bari, Italy. The isolation and identification procedures were conducted inthe Hygiene Section of the Department. Using conventional physiological and morphological methods (API systems), the strains were identified as *C. albicans* A18, *C. albicans* 10A12, C. albicans 810, *C. krusei* 31A29, *C. parapsilosis* 11A13, *C. parapsilosis* 1A1, *C. parapsilosis* 911, *C. parapsilosis 910* and *C. tropicalis* 810. All strains were grown and maintained on Sabouraud dextrose broth (Oxoid, Italy) at 37 °C.

### 4.2. Methods

#### 4.2.1. Gas Chromatography and Mass Spectrometry Equipment

Gas chromatographic analysis of EOs were performed on an Agilent 6890 N gas chromatograph equipped with a 5973 N mass spectrometer, provided with a HP-5 MS (5% phenylmethylpolysiloxane, 30 m, 0.25 mm i.d., 0.1 μm film thickness; J & W Scientific, Folsom) capillary column. The following temperature programmer was used: 5 min at 60 °C, then 4 °C/min to 220 °C, then 11 °C/min to 280 °C, held for 15 min, for a total run of 65 min. Injector and detector temperatures were 280 °C; the carrier gas was He; the flow rate was 1 mL/min; the split ratio was 1:50; the acquisition range was 29–400 *m/z* in electron-impact (EI) mode; and the ionization voltage was 70 eV.

#### 4.2.2. Compound Identification

For chemical characterization, EOs were diluted 1:100 in ethyl acetate and after filtration, 1 μL of each EO solution was injected into the GC-MS. Identification of the EOs’ components was done by comparison with authentic standards available in the authors’ laboratory. Qualitative analyses were carried out comparing the calculated Linear Retention Indices (LRIs) and Similarity Index Mass Spectra (SI/MS) for the obtained peaks with the analogous data from NIST 2017 and Adams 4th ed. (2007) databases. LRI of each compound was obtained by temperature programming analysis and was calculated in relation to a homologous series of *n*-alkanes (C7–C40) under the same operating conditions. LRI was calculated following the Van den Dool and Kratz equation [22] and compared with the Arithmetic Index (AI) from NIST 2017 database [26] and Adams, 4th ed. (Adams 2007) [25]. SI/MS were determined as reported by Koo et al. [27]. Component relative percentages were calculated based on GC peak areas without using correction factors. 

#### 4.2.3. Preparation of The Test Solution 

The EOs are solubilized in ethanol in 1:5 proportions and then diluted in Sabouraud added with tween 80. DSS should be solubilized in DMSO and subsequently in culture medium. 

#### 4.2.4. Antifungal and Susceptibility Tests

The antifungal activity of DSS was evaluated using a microdilution method as described by the Clinical and Laboratory Standards Institute (CLSI, M27-A3) [36]. Four double serial dilutions of the EOs were prepared following the same method used to evaluate the MIC described in our previous works [31,32]. Minimum inhibition concentration (MIC) determinations were made in triplicate. Two-fold serial dilutions of the NSAID were made with Yeast Malt Broth (YMB) to give concentrations ranging from 2.05 μg/mL to 0.03 μg/mL. MICs indicating the bacteriostatic effect of the DSS were obtained following incubation at 37 °C for 48 h. MICs were recorded as the lowest concentration of tested compound that completely inhibited fungal growth.

#### 4.2.5. Checkerboard Test

The checkerboard method was utilized to determine the synergistic, additive, or antagonistic effects of the combination of DSS and EOs. The tested dilutions were based on the MIC of the two substances. The combination of two compounds was synergistic when the FICI was ≤0.5, additive when the FICI was >0.5 and <1, and antagonistic when the FICI was >1. The test was performed using sterile 96-well microtiter plates containing DSS and EOs in two-fold serial concentrations. MICs were obtained following incubation at 37 °C for 48 h. 

Each test was performed in triplicate. A synergistic effect (FICI ≤ 0.5) between the two compounds is indicated as a concave curve, additive (FICI >0.5 and <1) interactions are represented by a straight line, and a convex curve indicates antagonism (FICI ≥ 1). This procedure allowed to evaluate with accurately the effect of synergism on the fungal growth.

### 4.3. Statistical Analysis

Every experiment for GC-MS has been replicated three times across three different days. The microbiological assays were performed for five times in five different days, giving an amount of 25 replicates.

Statistical analysis for microbiological assay (standard deviation, SD) and for chemical determination of structural equation modeling (SEM) was performed using Microsoft Excel. 

## 5. Conclusions 

The synergistic associations of drugs represent a valid approach in the antimicrobial therapies that have provided positive results in recent years. The rediscovery of natural products and their use in medical practice is quite recent and derives above all from the need to overcome the undesirable effects induced by conventional antimicrobials. The success of therapies based on natural products of plant origin has been scientifically evaluated with irrefutable research protocols in laboratory settings as well as in clinical practice. Our previous studies on EOs, based on the synergy with antibiotics, demonstrated the effectiveness of these associations by proposing the possibility of their possible therapeutic use. The data reported in this study underline that EOs, commonly sold and distributed, possess in vitro a decisive and strong action towards fungal *Candida* cells, belonging to different species in association with DSS, an NSAID whose activity against *Candida* spp. has been successfully confirmed. Results obtained indicate that small quantities of DSS and EO in association possess an excellent inhibitory capacity towards different strains of *Candida* spp. The effectiveness is conceivably the result of a multifactorial action, which escapes any resistance mechanisms that are now widespread and increasingly worrying. The in-vitro assays of these associations validate a sure efficacy against *Candida* infection, hither to never treated in scientifically proven research works. Further studies in the sector of EOs in association with NSAIDs are necessary to give us a better understanding of these phenomena related to fungal antibiosis from combinations of drugs and natural products. In this context our results may represent an interesting starting point for an alternative route to new synergistic antifungal therapies against fungal infections, overcoming the high cost of new drugs and the potential risk of antagonistic interactions. We are confident that these finding could represent a valid alternative to protect human health from infectious diseases.

## Figures and Tables

**Table 1:** Chemical composition of tested Essential Oils (Eos).

N	Components	LRI	AI	*Pelargonium graveolens*		*Mentha × piperita*		*Melaleuca alternifolia*	
				AREA% ± SEM	SI/MS	AREA% ± SEM	SI/MS	AREA% ± SEM	SI/MS
**1**	propanoic acid, ethylester	712	714	0.12 ± 0.012	86	0.11 ± 0.009	91		
**2**	α-thujene	924	926			0.04 ± 0.001	91	0.88 ± 0.020	91
**3**	α-pinene	933	933	0.59 ± 0.050	97	1.40 ± 0.010	97	2.14 ± 0.120	96
**4**	1-methyl-3-(2-methyl-1-propenyl)-cyclopentane	972	972	0.18 ± 0.050	80				
**5**	β-pinene	975	975			1.43 ± 0.500	96		
**6**	*trans*-carene	977	977			7.72 ± 2.110	91		
**7**	β-myrcene	987	988			0.13 ± 0.100	91		
**8**	2,6-dimethyl- 2,6-octadiene	991	990	1.01 ± 0.090	96				
**9**	3-octanol	995	995			0.13 ± 0.150	90		
**10**	*o*-cymene	1021	1021	0.10 ± 0.005	91	0.44 ± 0.050	95		
**11**	*p*-cymene	1025	1025					2.21 ± 0.990	95
**12**	(*Z*)−β-ocimene	1027	1027	0.10 ± 0.007	95				
**13**	3-isopropenyl-5,5-dimethyl-cyclopentene	1029	1028					1.68 ± 0.030	81
**14**	1,8-cineole	1031	1031			9.07 ± 2.090	98	2.13 ± 0.700	98
**15**	limonene	1033	1033	0.22 ± 0.040	94				
**16**	β-phellandrene	1035	1035			0.53 ± 0.010	91	0.23 ± 0.005	91
**17**	γ-terpinene ^a^	1058	1060			0.11 ± 0.002	96	17.18 ± 2.120	94
**18**	cis-linalool oxide	1070	1074	0.37 ± 0.001	90				
**19**	α−terpinolene	1081	1082					3.80 ± 0.020	96
**20**	linalol	1099	1098	4.68 ± 0.850	95				
**21**	rose oxide	1112	1112	1.67 ± 0.050	90				
**22**	*cis*-*p*-menth-2-en-1-ol	1119	1119					0.33 ± 0.005	93
**23**	*p*-menthone	1154	1154	2.19 ± 0.970	98				
**24**	*iso*-menthone ^a^	1164	1165	4.61 ± 1.700	98	23.99 ± 2.490	97		
**25**	menthol ^a^	1168	1169	0.14 + 0.003	91	35.60 + 1.760	91		
**26**	terpinen-4-ol ^a^	1174	1174					33.28 + 2.750	83
**27**	isopulegone	1177	1177			0.16 + 0.002	96		
**28**	*neo*-*iso*-menthol	1187	1188			9.33 + 1.100	96		
**29**	α-terpineol	1191	1190	0.45 + 0.090	80	0.59 + 0.010	87	2.84 + 0.350	86
**30**	citronellol ^a^	1220	1221	26.15 ± 3.260	98				
**31**	pulegone	1230	1236	0.11 ± 0.010	83	1.21 ± 0.400	98		
**32**	citral	1240	1240	0.70 ± 0.001	96				
**33**	piperitone	1250	1253			1.20 ± 0.030	96		
**34**	geraniol ^a^	1254	1254	11.70 ± 1.020	96				
**35**	citronellyl formate	1272	1275	6.85 ± 0.920	96				
**36**	geraniol formate	1280	1281	2.69 ± 0.100	86				
**37**	menthyl acetate	1294	1294			0.40 ± 0.005	91		
**38**	1,5,5-trimethyl-6-methylen-cyclohexene	1335	1338	0.33 ± 0.070	86				
**39**	citronellyl acetate	1358	1355	0.48 ± 0.050	94				
**40**	neryl acetate	1364	1367	1.47 ± 0.250	86				
**41**	isoledene	1376	1373					1.07 ± 0.090	95
**42**	β-bourbonene	1380	1382	1.80 ± 0.140	95				
**43**	langifolene	1405	1405					0.12 ± 0.009	90
**44**	1-H-indene-1-ethylideneocta hydro-7a-methyl-(1z,3a.a,7a.b)	1410	1409	0.64 ± 0.040	95				
**45**	α-guajene	1413	1413	0.39 ± 0.001	98				
**46**	(*E*)-caryophyllene	1420	1419	1.63 ± 0.020	99	2.13 ± 0.950	99	1.09 ± 0.013	99
**47**	β-copaene	1428	1428	1.06 ± 0.015	99				
**48**	neryl propionate	1430	1430	0.15 ± 0.023	80				
**49**	aromadendrene	1440	1440	0.70 ± 0.090	99			4.41 ± 1.090	99
**50**	citronellyl propionate	1445	1445	1.06 ± 0.030	64				
**51**	humulene	1452	1452	0.38 ± 0.001	97	0.12 ± 0.090	95	0.20 ± 0.001	97
**52**	α−amorphene	1455	1455	0.87 ± 0.025	96			0.32 ± 0.015	99
**53**	(*E*)-β-farnesene	1459	1459			0.10 ± 0.080	95		
**54**	γ−muurolene	1474	1474	0.73 ± 0.055	90			0.15 ± 0.090	83
**55**	*epi*-bicyclosesquiphellandrene	1482	1482					1.00 ± 0.078	87
**56**	4,11-selinadiene	1483	1485	0.18 ± 0.074	92				
**57**	δ−selinene	1490	1493	0.17 ± 0.007	97				
**58**	ledene	1495	1495					3.93 ± 1.670	95
**59**	δ−cadiene	1524	1524					2.98 ± 0.430	95
**60**	α-panasinsene	1527	1527					0.16 + 0.009	93
**61**	α−calacorene	1542	1540	0.11 ± 0.001	91				
**62**	geranyl butyrate	1554	1555	1.49 ± 0.012	96				
**63**	*neo*-isolongifolene	1558	1558	0.18 ± 0.004	83				
**64**	spathulenol	1578	1578	0.35 ± 0.002	91			0.11 ± 0.008	99
**65**	phenylethyl tiglate	1584	1584	1.48 + 0.015	90				
**66**	globulol	1585	1585					0.54 ± 0.001	98
**67**	caryophyllene oxyde	1592	1592			0.28 ± 0.070	95		
**68**	γ−eudesmol ^a^	1620	1619	7.02 ± 2.050	99				
**69**	(*E*)-citronellyl tiglate	1665	1667	0.38 ± 0.009	91				
**70**	geranyl tiglate	1701	1700	1.57 ± 0.080	91				
	**% Characterized**			**89.40**		**96.22**		**82.78**	
	Others			10.60		3.78		17.22	

^a^: standard compounds. Linear retention index (LRI) on HP-5MS column was experimentally determined using a homologous series of C7–C40 alkanes standard mixture [24]. Arithmetic index (AI) was taken from Adams 4th Ed. (2007) [25] and/or the NIST 2017 Database [26]. Similarity index/mass spectrum (SI/MS) was compared with data reported on NIST 2017 Database and were determined as reported by Koo et al. [27], and Wan et al. [28]. Relative percentage values are means of three determinations with a structural equation modeling (SEM) in all cases below 10%.

**Table 2:** Antifungal activity of *M. piperita* Essential Oil (EO) and Diclofenac Sodium Salt (DSS) on different *Candida* strains.

Strains	EO	DSS	Synergism		
	MIC ^a^ ± SD	MIC ^a^ ± SD	DSS μg/mL ^b^	EO μg/mL ^c^	FICI ^d^
*C. albicans ATCC 10231*	1.00 ± 0.480	1.02 ± 0.350	0.51	0.05	0.30
*C. albicans ATCC 90028*	1.00 ± 0.450	1.02 ± 0.370	0.51	0.05	0.30
*C. glabrata* ATCC 15126	1.00 ± 0.500	2.05 ± 0.790	0.10	0.51	0.30
*C. tropicalis* ATCC 750	1.00 ± 0.450	1.02 ± 0.350	0.20	0.06	0.22
*C. kefyr* ATCC 204093	0.25 ± 0.020	2.05 ± 0.800	0.20	0.13	0.30
*C. krusei* ATCC 6258	0.50 ± 0.030	1.02 ± 0.390	0.06	0.31	0.30
*C. albicans* A18	1.00 ± 0.080	2.05 ± 0.500	0.10	0.51	0.30
*C. albicans* 10A12	0.50 ± 0.030	1.02 ± 0.310	0.20	0.13	0.30
*C. albicans* 810	1.00 ± 0.20	1.02 ± 0.250	0.20	0.13	0.30
*C. krusei* 31A29	1.00 ± 0.310	2.05 ± 0.620	0.41	0.25	0.30
*C. parapsilosis* 11A13	1.00 ± 0.060	1.02 ± 0.200	0.05	0.51	0.30
*C. parapsilosis* 1A1	0.50 ± 0.020	2.05 ± 0.830	0.41	0.13	0.30
*C. parapsilosis* 911	0.25 ± 0.060	1.02 ± 0.270	0.10	0.06	0.22
*C. parapsilosis 910*	0.12 ± 0.040	1.02 ± 0.410	0.10	0.03	0.22
*C. tropicalis* 810	0.50 ± 0.020	1.02 ± 0.450	0.10	0.12	0.22

^a^: MIC minimal inhibitory concentration (%v/v for EO; μg/mL for DSS); ^b^: concentration of DSS in the mixture; ^c^: concentration of essential oil in the mixture; ^d^: FICI: fractional inhibitory concentration index; DSS: Diclofenac Sodium Salt; EO: Essential Oil; SD: Standard Deviation.

**Table 3:** Antifungal activity of *P. graveolens* Essential Oil (EO) and Diclofenac Sodium Salt (DSS) on different *Candida* strains.

Strains	EO	DSS	Synergism		
	MIC ^a^ ± SD	MIC ^a^ ± SD	DSS μg/mL ^b^	EO μg/mL ^c^	FICI ^d^
*C. albicans ATCC 10231*	0.12 ± 0.021	1.02 ± 0.350	0.10	0.03	0.23
*C. albicans ATCC 90028*	0.25 ± 0.017	1.02 ± 0.370	0.20	0.06	0.30
*C. glabrata* ATCC 15126	0.25 ± 0.015	2.05 ± 0.790	0.20	0.06	0.23
*C. tropicalis* ATCC 750	0.12 ± 0.013	1.02 ± 0.350	0.10	0.03	0.23
*C. kefyr* ATCC 204093	0.12 ± 0.014	2.05 ± 0.800	0.10	0.06	0.30
*C. krusei* ATCC 6258	0.50 ± 0.021	1.02 ± 0.390	0.20	0.12	0.30
*C. albicans* A18	0.25 ± 0.021	2.05 ± 0.500	0.41	0.06	0.33
*C. albicans* 10A12	0.12 ± 0.012	1.02 ± 0.310	0.20	0.03	0.30
*C. albicans* 810	0.12 ± 0.010	1.02 ± 0.250	0.10	0.03	0.23
*C. krusei* 31A29	0.50 ± 0.084	2.05 ± 0.620	0.41	0.12	0.30
*C. parapsilosis* 11A13	0.50 ± 0.082	1.02 ± 0.200	0.20	0.06	0.30
*C. parapsilosis* 1A1	0.25 ± 0.070	2.05 ± 0.830	0.41	0.06	0.26
*C. parapsilosis* 911	0.25 ± 0.072	1.02 ± 0.270	0.20	0.03	0.30
*C. parapsilosis 910*	0.25 ± 0.079	1.02 ± 0.410	0.05	0.12	0.30
*C. tropicalis* 810	0.25 ± 0.052	1.02 ± 0.450	0.10	0.12	0.35

^a^: MIC minimal inhibitory concentration (%v/v for EO; μg/mL for DSS); ^b^: concentration of DSS in the mixture; ^c^: concentration of essential oil in the mixture; ^d^: FICI: fractional inhibitory concentration index; DSS: Diclofenac Sodium Salt; EO: Essential Oil; SD: Standard Deviation.

**Table 4:** Antifungal activity of *M. alternifolia* Essential Oil (EO) and Diclofenac Sodium Salt (DSS) on different *Candida* strains.

Strains	EO	DSS	Synergism		
	MIC ^a^ ± SD	MIC ^a^ ± SD	DSS μg/mL ^b^	EO μg/mL ^c^	FICI ^d^
*C. albicans ATCC 10231*	0.50 ± 0.021	1.02 ± 0.350	0.20	0.25	0.45
*C. albicans ATCC 90028*	0.50 ± 0.020	1.02 ± 0.370	0.10	0.13	0.23
*C. glabrata* ATCC 15126	0.50 ± 0.012	2.05 ± 0.790	0.20	0.13	0.23
*C. tropicalis* ATCC 750	0.50 ± 0.015	1.02 ± 0.350	0.20	0.03	0.23
*C. kefyr* ATCC 204093	1.00 ± 0.112	2.05 ± 0.800	0.82	0.51	//
*C. krusei* ATCC 6258	0.50 ± 0.025	1.02 ± 0.390	0.40	0.25	//
*C. albicans* A18	0.25 ± 0.001	2.05 ± 0.500	0.82	0.15	0.43
*C. albicans* 10A12	0.50 ± 0.025	1.02 ± 0.310	0.20	0.25	0.45
*C. albicans* 810	0.50 ± 0.022	1.02 ± 0.250	0.40	0.06	0.45
*C. krusei* 31A29	0.50 ± 0.027	2.05 ± 0.620	0.82	0.25	//
*C. parapsilosis* 11A13	0.50 ± 0.023	1.02 ± 0.200	0.05	0.25	0.30
*C. parapsilosis* 1A1	0.50 ± 0.030	2.05 ± 0.830	0.20	0.25	0.35
*C. parapsilosis* 911	0.50 ± 0.042	1.02 ± 0.270	0.05	0.25	0.30
*C. parapsilosis 910*	0.50 ± 0.050	1.02 ± 0.410	0.40	0.03	0.43
*C. tropicalis* 810	0.50 ± 0.045	1.02 ± 0.450	0.20	0.25	0.45

^a^:MIC minimal inhibitory concentration (%v/v for EO; μg/mL for DSS); ^b^: concentration of DSS in the mixture; ^c^: concentration of essential oil in the mixture; ^d^: FICI: fractional inhibitory concentration index; DSS: Diclofenac Sodium Salt; EO: Essential Oil; SD: Standard Deviation.

## Data Availability

The date is available in the manuscript.

## Abbreviations

DSS	Diclofenac Sodium Salt
Eos	EssentialOils
GC	Gas Chromatography
MS	Mass Spectrometer
SEM	Structural Equation Modeling
LRI	Linear Retention Indices
AI	Arithmetic Index
SI/MS	Similarity Index/Mass Spectra
MIC	Minimal Inhibitory Concentration
FICI	fractional inhibitory concentration
